# Conceptual Model of Emergency Department Utilization among Deaf and Hard-of-Hearing Patients: A Critical Review

**DOI:** 10.3390/ijerph182412901

**Published:** 2021-12-07

**Authors:** Tyler G. James, Julia R. Varnes, Meagan K. Sullivan, JeeWon Cheong, Thomas A. Pearson, Ali M. Yurasek, M. David Miller, Michael M. McKee

**Affiliations:** 1 Department of Family Medicine, School of Medicine, University of Michigan, 1018 Fuller St., Ann Arbor, MI 48104, USA; mmmckee@med.umich.edu; 2 Department of Health Education and Behavior, University of Florida, Florida Gym Room 5, P.O. Box 118210, Gainesville, FL 32611, USA; jwcheong@ufl.edu (J.C.); a.yurasek@ufl.edu (A.M.Y.); 3 Department of Health Services Research, Management, and Policy, University of Florida, P.O. Box 100185, Gainesville, FL 32610, USA; jrvarnes@ufl.edu; 4 Independent Researcher, Gainesville, FL 32601, USA; msully94@ufl.edu; 5 Department of Epidemiology, University of Florida, P.O. Box 100231, Gainesville, FL 32610, USA; tapearson@ufl.edu; 6 School of Human Development and Organizational Studies in Education, University of Florida, P.O. Box 117047, Gainesville, FL 32611, USA; dmiller@coe.ufl.edu

**Keywords:** deaf, hard of hearing, hearing loss, critical review, emergency department, health behavior, conceptual model

## Abstract

Deaf and hard-of-hearing (DHH) populations are understudied in health services research and underserved in healthcare systems. Existing data indicate that adult DHH patients are more likely to use the emergency department (ED) for less emergent conditions than non-DHH patients. However, the lack of research focused on this population’s ED utilization impedes the development of health promotion and quality improvement interventions to improve patient health and quality outcomes. The purpose of this study was to develop a conceptual model describing patient and non-patient (e.g., community, health system, provider) factors influencing ED utilization and ED care processes among DHH people. We conducted a critical review and used Andersen’s Behavioral Model of Health Services Use and the PRECEDE-PROCEED Model to classify factors based on their theoretical and/or empirically described role. The resulting Conceptual Model of Emergency Department Utilization Among Deaf and Hard-of-Hearing Patients provides predisposing, enabling, and reinforcing factors influencing DHH patient ED care seeking and ED care processes. The model highlights the abundance of DHH patient and non-DHH patient enabling factors. This model may be used in quality improvement interventions, health services research, or in organizational planning and policymaking to improve health outcomes for DHH patients.

## 1. Introduction

The United States is facing increasing rates of emergency department (ED) utilization paired with the closure of EDs nationwide [1,2]. ED utilization, compared to other sources of care, is more prevalent for specific priority populations (e.g., publicly insured, limited English proficient (LEP), and racially, ethnically, and linguistically minoritized populations) [3,4,5,6]. Linking patients to more continuous sources of care such as primary and specialty care would lead to better patient outcomes and reduced public health expenditures [7,8]. However, it is necessary to first understand what influences ED care-seeking among these priority populations, to further investigate disparities in health service utilization and delivery and justify the implementation of quality improvement programs.

The DHH community is one such priority population experiencing widespread determinants of ED utilization that are understudied [9]. This gap delays the field from holistically and deeply understanding the unique factors that influence DHH patient ED utilization and care quality and consequently represents a critical barrier to achieving health equity for DHH patients. In order to catalyze health equity efforts for this population, and accomplish national health objectives in the U.S. [10,11], additional research must be conducted to identify facilitators, barriers, and reasons for ED use. 

Approximately 17% of the U.S. population has an identifiable hearing loss [9,12]. The DHH population is heterogeneous and can be characterized by various factors, including the age of onset of hearing loss, type of loss, language modality, and cultural affiliation; each influences antecedents to healthcare utilization [9,13]. DHH sign language users represent a linguistic and cultural minority group who, in the U.S., predominately use American Sign Language (ASL) to communicate [14]. In comparison, DHH spoken-language users are typically older adults with age-related hearing loss. Thus, they may have relatively better English proficiency and more substantial funds of information than DHH ASL-users and possess more skills to navigate healthcare effectively. 

DHH individuals experience a variety of risk factors for ED utilization and evidence suggests that DHH patients are, in fact, more likely to use the ED than their non-DHH English-speaking counterparts [15,16]. However, the complexity of ED utilization outcomes among DHH patients has not been further explored; this impedes efforts to develop quality improvement, health education, and healthcare navigation interventions to ensure DHH patients are appropriately and effectively using healthcare services. The field requires a sophisticated, evidence-based, and theory-informed understanding of DHH ED utilization. Thus, the aim of the present study was to develop a conceptual model describing ED utilization among DHH patients applying commonly used models in health services research and health promotion program planning. The presented model can be used for public health program development, quality improvement programs, and research focused on DHH patient health. 

## 2. Materials and Methods

### 2.1. Conceptual Models and Critical Reviews

A conceptual model is “a diagram of proposed causal linkages among a set of concepts believed to be related to a health problem” [17], that seeks to synthesize available evidence to guide research and practice [18,19,20]. Although there is no standardized method for developing conceptual models, previous research in the health sciences has employed qualitative studies, narrative reviews, and systematic reviews to assist in conceptual model development. In health education and promotion, Earp and Ennett [17] recommend a conceptual model development process including identifying endpoints of interest, starting with existing conceptual frameworks and theories, and then identifying concepts based on the empirical literature and researcher knowledge. After consultation with academic librarians specializing in the health sciences, a search of MEDLINE (PubMed) and Web of Science (on 28 May 2019) indicated that a systematic review would be unsuccessful: yielding only four relevant empirical articles [15,21,22,23,24]. Therefore, we were advised to implement a “critical review.” A critical review is a non-systematic review that focuses on integrating “conceptual innovation” from diverse sources (e.g., peer-reviewed, gray literature, court cases) related to the research problem [25], as opposed to a systematic review. Whereas systematic reviews serve as an endpoint to a research question, critical reviews serve as a “launchpad” or a starting point, and typically result in a research hypothesis or conceptual model [25]. 

### 2.2. Definining Primary Endpoints

Endpoints, or outcomes, in conceptual models are typically related to intervention targets of health education/promotion programs [17]. For this model, we chose to focus on the following primary outcomes: ED utilization, ED length of stay (LOS), and revisiting the ED. 

#### 2.2.1. ED Utilization

Evidence to date indicates that DHH patients use EDs more frequently than their non-DHH counterparts. Community-sampled data from Rochester, NY (home to one of the largest per capita DHH ASL-using populations in the U.S.) indicates 16.2% of DHH ASL-users people reported using the ED two or more times in the past year, as reported in a survey conducted in 2013; a rate almost 2.5 times higher than the general U.S. population [23]. In Florida, a pilot community-engaged survey in 2018 found that 55.6% of DHH ASL-users reported using the ED in the past 12 months [26]. To our knowledge, there has only been one published medical record review study evaluating differences in ED utilization among DHH ASL-users and non-DHH English-speaking patients (in Rochester): when adjusting for demographic variables, DHH ASL-users had approximately 2.0 times higher odds than non-DHH English-speaking patients of using the ED between 2009 and 2012 [15]. Data specific to the DHH English-speaking population also indicate higher risk. Among patients 50 years and older, DHH English-speaking patients who have untreated hearing loss have a 16.9% increased risk of ED utilization than their non-DHH counterparts [16]. Notably, the field lacks an understanding regarding the conditions for which DHH patients seek ED care. McKee et al. (2015) qualitatively described that almost half (48%) of DHH ASL-users’ ED encounters had low condition acuity, as opposed to 35% of non-DHH English-speaking patients. However, this represents an important gap in the literature that warrants additional study. 

#### 2.2.2. ED Length of Stay (LOS)

Increasing ED utilization can lead to situations where demand for ED use outweighs the supply of resources. The National Academy of Medicine recognizes ED burden and overcrowding as a critical public health issue [27] as it negatively impacts patients’ health, including patients leaving the ED before being seen, experiencing delays in patients being treated, and increasing the risk of medical errors [28]. 

An outcome related to, but not a direct measure of, ED overcrowding is ED LOS [29]. The Centers for Medicare and Medicaid Services [30] recognize ED LOS as a Clinical Quality Measure of the patient’s experience of care. For this study, ED LOS is defined as the time from ED arrival to departure for patients discharged home or admitted to an inpatient unit from the ED (also known as “throughput time”). Increased ED LOS can occur when the ED is crowded which may be due to boarding issues in trying to admit patients such as hospital bed shortages, delays in diagnostic or specialty care, day and time of ED utilization, and patient condition factors (e.g., condition acuity) [29,31,32]. Longer LOS is associated with worse patient experiences [33] and worse health outcomes. For example, longer ED LOS may cause some patients to leave the ED without being seen [34]; and ED crowding, generally, increases ambulance diversion [35,36]. Thus, identifying factors associated with ED LOS is critical to guide intervention development for improving health delivery (e.g., reducing LOS for patients, and improve wait times). Currently, the field lacks information regarding ED LOS among DHH patients. One study conducted in Rochester, NY, assessed ED throughput time among DHH ASL-users in addition to patients who use Spanish to communicate; in this study, patients who used an interpreter had longer ED LOS than patients who did not use an interpreter [37]. Although ED LOS is attributable to factors primarily outside of ED providers’ control, as discussed in the proposed model, there may be DHH specific factors that contribute to longer ED LOS. 

#### 2.2.3. ED Revisit

An ED revisit occurs when a patient is discharged from the ED and then returns to the ED within a specified timeframe. Revisits may occur for a variety of reasons including patients experiencing major side-effects of treatment or additional symptoms of their initial condition, poor patient adherence to treatment plans (for reasons that exist both within and externally to the patient), and difficulties navigating the healthcare system [38]. The U.S. Agency for Healthcare Research and Quality (AHRQ) has identified that frequent ED revisits or ED revisits within acute timeframes (e.g., 9 days [39]) warrant further investigation as the revisit may indicate ED discharge failure [40]. ED discharge failure can occur when patients do not adequately understand their diagnosis or treatment plan, or do not have access to resources (tangible or behavioral) necessary to navigate healthcare post-discharge. There is little information regarding the occurrence of revisits, and discharge failure, among DHH patients. In Rochester, NY, 29.3% of DHH ASL-using patients had used the ED more than one time over a 36 month period, compared to 10.4% of non-DHH English-speaking patients [15]; however, we lack information on the proportion of these encounters that were acute revisits (e.g., within 9 days) in addition to the specific reason for the revisit. 

### 2.3. Conceptual Basis

This conceptual model is grounded in frameworks commonly used in health services research and health promotion: the social-ecological model (SEM) [41], Andersen’s Behavioral Model of Health Services Use [42], and the PRECEDE-PROCEED Model (PPM) [43]. The purpose of this was two-fold: (1) using existing theory is a best practice in developing conceptual frameworks [17,19], and (2) this enables an expansion of the literature using familiar terminology (reducing barriers to using this model), but specific to DHH patient populations.

#### 2.3.1. Social-Ecological Model (SEM)

It is well understood that individual-level factors do not solely influence health behavior and healthcare utilization; thus, the field must consider multiple levels of influence to better understand DHH ED utilization. Ecological models provide a framework for understanding factors within each level of influence so the field can appropriately determine intervention and measurement opportunities. Studies applying the SEM use a varying number of levels of influencing factors [41]. For this model, we focused on the following four levels: (1) individual; (2) interpersonal; (3) community, organization, and provider; and (4) federal, state, and local policy levels. ED providers are included at the community and organization level because we conceptualize a highly dynamic relationship between provider behavior and beliefs and organization culture [44].

#### 2.3.2. Andersen’s Behavioral Model of Health Services Use and the PRECEDE-PROCEDE Model

Andersen’s Behavioral Model of Health Service Use [42] is a health services research model that emphasizes the relation between individual and contextual factors in health service utilization, and has been applied in ED research [45,46]. Originally developed in 1968, the model describes the influence of contextual factors—including health organization, provider, and community characteristics—on individual-level factors and healthcare utilization. Within both the contextual and individual components of the model, there are three primary constructs: predisposing factors, enabling factors, and need. The primary purpose of the Andersen Model is to describe factors associated with these constructs to develop appropriate research questions and inform statistical model-building approaches. Inspired by the Andersen Model, the PRECEDE-PROCEED model (PPM) was developed for planning and evaluating health education and promotion interventions [43,47]. Like the Andersen Model, the PPM includes predisposing and enabling constructs with similar definitions; however, the PPM also includes reinforcing constructs. As a planning model, PPM provides a step-by-step approach to addressing quality of life issues through an epidemiological, and ecological and educational diagnosis. Through this process, the PPM elucidates the relationship between social and non-social factors (called predisposing, enabling, and reinforcing factors) and epidemiologic factors including environmental conditions of living, genetic factors, and individual health behavior on health outcomes. 

For this review, we assessed predisposing, enabling, need, and reinforcing characteristics when evaluating potential relations between model constructs and ED outcomes. 

**Predisposing.** Predisposing factors represent conditions that influence people to use or not use services; however, they are not directly responsible for health or healthcare utilization behavior. Predisposing constructs are defined in the PPM as “a person’s or population’s knowledge, attitudes, beliefs, values, and perceptions that facilitate or hinder motivation for change” [43]; this definition aligns with the Andersen Model. At the contextual-level, Andersen defines predisposing factors as including population demographics, beliefs or underlying community values and norms, the individual level, demographic factors and “biological imperatives,” social factors and social networks, and health beliefs and attitudes [42]. 

**Enabling.** Enabling conditions directly facilitate or impede healthcare service utilization. Although the Andersen Model and PPM agree that enabling factors occur at both the individual and broader environmental levels of the SEM, PPM considers enabling factors as primarily environmental facilitators to behavior [43]. Contextually, these factors include community policies, the distribution and availability of health services within a geographic area, and per capita income [42]. Individually, enabling conditions include skills and resources including health insurance, money to pay for healthcare, transportation, and time-waiting for care [42,43]. 

**Need.** ‘Need’ delineates a community’s or individual’s need to engage in healthcare services. At the contextual-level, need is related to the physical environment such as housing and air quality, and rates of injury and death. At the individual level, need is characterized into two sub-constructs: perceived need and evaluated need. Perceived need is how individuals view their health and quality of life and include pain perceptions and perceived severity. Evaluated need, however, is based on judgment and physical examination from medical professionals and includes vital signs and diagnoses. Importantly, the Andersen Model states that perceived need is a social phenomenon and should be “largely explainable by social characteristics and health beliefs” [42]. 

**Reinforcing.** In the PPM, reinforcing constructs are related to an individual receiving positive or negative feedback for engaging or not engaging in a behavior [43]. Reinforcement primarily occurs outside of the individual level of the SEM and includes social rewards and punishment from friends, family, community members, and medical providers, but can also include non-social (i.e., physiological) consequences (e.g., coughing the first time a person smokes a cigarette) [43]. Although social rewards are conceptualized as predisposing factors in the Andersen Model, we applied the PPM’s conceptualization of social support for this model’s development, not the Andersen Model’s. 

### 2.4. Literature Search

Following the general methods of critical reviews, the literature search strategy was not systematic [25]. As a starting point, we started with the eight articles identified through the systematic search focusing on DHH ED utilization. Then, we forward and back-cited articles to identify conceptual innovation, and integrated literature focusing on populations with similar ED utilization patterns and communication complexities that are, relatively, better studied (e.g., limited English proficient (LEP) and older adult populations). Further, we looked outside of the peer-reviewed literature, and included theses and dissertations, expert position statements (e.g., from national organizations), and judicial documents. The inclusion criteria were: (1) published after 1980; (2) published in English or ASL; and (3) accessible through print journals, library databases or websites, or library-loan programs. 

## 3. Proposed Conceptual Model and Literature Support

**Figure 1:**
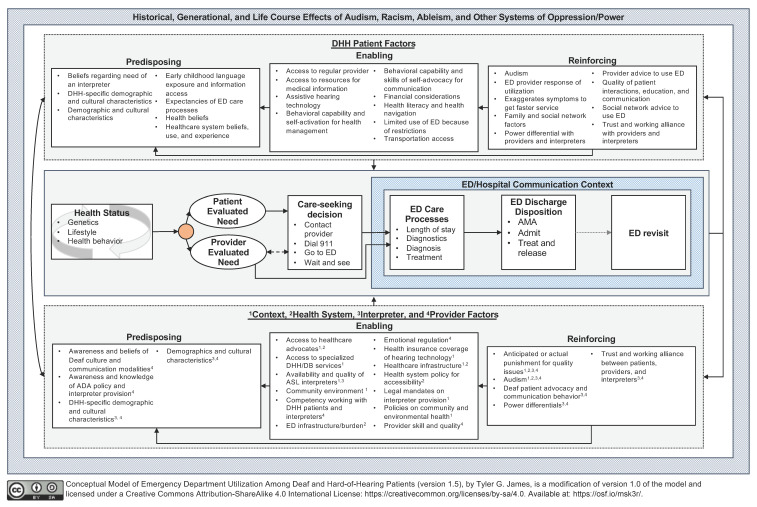
Conceptual model of emergency department utilization among deaf and hard-of-hearing patients.

Figure 1 provides a visual representation of the proposed *Conceptual Model of Emergency Department Utilization Among Deaf and Hard-of-Hearing Patients*, describing factors (defined in Table A1 and Table A2) related to DHH patient ED utilization and ED care processes. The model posits that the central outcomes (i.e., lifestyle and health behavior, need-based factors, decision-making for care-seeking, and ED care processes) are affected by social, behavioral, and biological antecedents at the DHH patient and non-patient (e.g., healthcare provider, interpreter, health system, society) levels. The purpose of this section is to provide an overarching explanation of how these antecedents influence central outcomes. This explanation is non-exhaustive for narrative parsimony and to avoid reducing the dimensional complexity and interactive relations between predisposing, enabling, and reinforcing constructs. For example, health literacy is a multifaceted construct that interacts across the entire model but is only discussed in depth in one section. 

### 3.1. Health Status: Genetics, Lifestyle, and Health Behavior

The first part of the model states that a DHH person’s health status is indicated by their lifestyle, health behavior, and genetics. A cyclical relation among them is specified due to the potential of gene-environment interactions that, in the presence of behavioral or physical environmental factors, may be deleterious or beneficial to a DHH person’s health. 

**Genetic and congenital factors.** The PPM and Andersen Model agree that genetic factors are important characteristics when considering health behavior and health status. When considering the DHH population, both genetic and congenital etiologies of hearing loss may predispose people to additional conditions. There are over 400 genes that may lead to being DHH, but these may also lead to other health conditions [9]; for example, mutations of the gene *GJB2*, which leads to connexin-26 mutations, one of the most common causes of non-syndromic hearing loss [48], are also associated with syndromic deafness and skin disorders [49]. In addition, in the early 1960s, German Measles (or rubella) caused over 20,000 cases of Congenital Rubella Syndrome (CRS) [50]. CRS commonly caused congenital sensorineural hearing loss, leading to many DHH individuals who are now in their late 50s/early 60s. As adults with CRS, these people face an increased risk of diabetes, glaucoma and cataracts, and increased blood pressure [51,52]. Thus, genetic and congenital factors (e.g., that lead to a person becoming DHH or not) may predispose a DHH individual to have worse health and, therefore, be more likely to need healthcare. 

**Fundamental causes to social determinants of health.** A “fundamental cause” [53,54,55] affecting a DHH person’s social determinants of health and resulting behavior, at the individual and contextual levels, can be attributed to a system of oppression known as audism. Audism is (1) “the notion that one is superior based on one’s ability to hear or behave in the manner of one who hears,” (2) “a system of advantage based on hearing ability,” and (3) “a metaphysical orientation that links human identity with speech” [56]. Prejudice and discrimination rooted in audism are a daily experience for DHH people. For example, audism is at work, at the interpersonal-level, when people (1) think being DHH is a tragedy, (2) discriminate against DHH people seeking employment, and (3) hold negative perceptions of people who do not use spoken language. The influential role of audism in the lives of DHH people can be seen in the faulty scientific, medical, and social philosophies across several fields, rooted in the eugenics movement [57], which have led to a systemic cascade of events that may negatively impact a DHH child’s language development. This cascade includes, but is not limited to: (1) a lack of information provided to parents of DHH children on sign language, (2) discouraging sign language use with DHH children, and (3) denying medical services if a parent attempts non-English speaking/hearing modalities [58,59]. Audism, and its resulting systems and philosophies of oppression, has caused widespread language deprivation among people who become DHH early in life. 

Language deprivation is defined as “delayed and/or absent exposure to an accessible first-language foundation” [60] and is, therefore, a preventable early childhood factor with lifelong consequences for any child whether or not they are DHH. The process of language deprivation occurs when a child is not exposed to accessible language within the critical period of language development. In a retrospective study, Hall et al. found that parental hearing status was a significant factor of comprehending indirect family communication: DHH ASL-users with non-DHH parents were less likely to comprehend indirect communication than those with at least one DHH parent [60]. This finding is noteworthy as most DHH children are born to non-DHH parents who do not use signed language at home [61,62]. Therefore, DHH children are at a much higher risk of being language deprived than non-DHH children. (Incidental learning is learning that is unplanned or unintended. For example, learning that occurs when watching the television or participating in a family dinner conversation. For further relevance to the experience of DHH children, see Hall et al. (2018) [59]) The lack of incidental learning and indirect familial communication leads to horizontal learning within the DHH ASL-using community: DHH ASL-users are more likely to receive information, including health information, from their DHH peers than they are from their parents [63,64,65]. 

The effect of a DHH person being language deprived and experiencing communication neglect is seemingly limitless. A DHH person who experiences language deprivation syndrome may have a higher likelihood of self-injury or suicide behavior, limited understanding of abstract concepts, difficulty with learning and emotional regulation, and lower funds of information [58]. Language deprivation can impact all aspects of an affected person’s life including educational and employment opportunities, physical and mental health, interpersonal relationships, and legal consequences. The impact of language deprivation, however, is “not the fault of deaf people; nor is (language deprivation) an evitable consequence of deafness” [58]. As noted by Caselli et al. [66], deafness is an audiological diagnosis while language deprivation is an “acquired consequence” of limited language exposure. A DHH child may have a strong language foundation; a non-DHH child may be language deprived. 

Audism and language deprivation, conceptualized as fundamental causes, impact the social determinants of health [11] and are the primary cause of DHH peoples’ hurdles to economic stability and education. (We have attempted to be parsimonious in explaining the complex topics and systems related to audism and language deprivation, and we are at risk of oversimplifying the role of these fundamental causes. As mentioned, the effects of language deprivation and audism are far reaching beyond just education and economic stability.) Exposure to an accessible language, such as ASL, can serve as a bridge to bilingual language development [67,68]. Without access to a language base, DHH people are at risk for limited majority-language (e.g., English in the U.S.) proficiency; the average English reading proficiency level among DHH ASL-users is similar to non-DHH 6th graders [69]. Limited English proficiency in an English-dominated society leads to stigmatization, information marginalization, and reduced economic opportunities. For example, compared to non-DHH people, DHH people in the U.S. are less likely to finish high school, and matriculate and finish college [70] and are more likely to be un-/underemployed [71]. The subsequent economic barriers have detrimental implications on social risk factors, including healthcare affordability and access to health-promoting resources (e.g., food security [72,73]). 

**Health behavior and health status.** Overall, the social determinants of health more likely to affect DHH people lead to social risk factors and social needs associated with health-compromising behavior. DHH people experience a variety of health inequities including reporting higher rates of substance use [23,74], worse mental health status and a higher likelihood of engaging in suicidal behavior [75,76], and greater health comorbidities [16,77]. There are also related disparities in socio-behavioral antecedents. For example, DHH ASL-users have almost seven times higher odds of having inadequate health literacy than their non-DHH English-speaking counterparts [78], and have limited knowledge related to common health conditions including cardiovascular disease, HIV/AIDS, and cancer [79,80,81,82]. 

These inequities do not reflect a lack of interest in health education/promotion. For example, almost half of DHH college students are interested in suicide prevention information [75] and there has been an increase in community-engaged DHH health research nationwide [23,83,84,85]. A primary barrier to health-promoting behavior is the lack of accessibility of conventional health education/promotion materials [86] and usability of health content accessible in ASL [87]. This leads to added burden among DHH people to access health-promoting information. 

Another factor closely related to health behavior and lifestyle is the context or environmental health conditions (e.g., housing, water, and air quality), which are inextricably linked to other fundamental causes (e.g., racism) and social determinants of health (e.g., economic stability). The role of environmental health is directly applicable to the DHH population. The Flint, Michigan region of the U.S. has a large DHH ASL-using community due to the proximity of the Michigan School for the Deaf and the historically industrial jobs for DHH people [14]. During the ongoing Flint water crisis (starting in 2014), the Michigan School for the Deaf reported lead in the school’s water [88]. The introduction of lead in the community’s water system worsened the health status of the Flint community [89], and likely also impacted the DHH community living and working in Flint. 

**Healthcare utilization.** In the model, regular care is considered a form of health behavior; this includes wellness and screening visits, and treatment of chronic conditions with primary and specialist providers. DHH people have varying rates of healthcare utilization. 

In 2018, a higher prevalence of DHH English-speakers than DHH ASL-users in Florida reported receiving a routine check-up in the past 12 months [74]. Nationally, among those insured, approximately 11% more non-DHH people have a usual provider than DHH ASL-users [90]. Breast cancer and prostate cancer screening uptake are not different among DHH ASL-users and their non-DHH counterparts [91,92]; however, DHH ASL-using men report feeling less engaged in shared-decision making with providers when receiving prostate cancer screenings [91]. 

Healthcare utilization among DHH people, like their non-DHH counterparts, is related to factors such financial resources and trust in providers. With respect to the affordability of healthcare, more DHH ASL-users [23] and more DHH English-speakers [74] report forgoing healthcare due to cost than non-DHH English-speakers. DHH people also experience difficulties with health insurance literacy and navigation [65]. In other populations, low health insurance literacy is associated with less frequent healthcare utilization [93]. 

A more pronounced difference between DHH and non-DHH patients’ healthcare utilization is related to the communication environment that influences patient–provider relationships and healthcare decisions. As described in the forthcoming sections, communication with healthcare providers and power dynamics profoundly impacts DHH patient health outcomes. Communication with providers is an essential consideration for engaging in routine healthcare. Nationally, DHH ASL-users are more likely to have a regular provider than those who prefer English or both English and ASL [90]. When DHH patients access healthcare they may encounter inaccessible patient–provider communication, compromising power and trust dynamics with providers [94]. For example, 37% of DHH ASL-users in Florida reported that they had been denied an interpreter at a medical facility in the year prior to a community-engaged survey [74]. At the provider level, nurses support the notion that they lack knowledge of working with DHH patients and believe that deferring to a non-DHH friend or family member for medical communication is appropriate [95]. However, using a non-DHH friend or family member as an impromptu “interpreter” is detrimental to patient communication and patient privacy rights [94]. DHH ASL-users report difficulty finding primary care providers who will provide accessible, patient-centered communication and seek recommendations from DHH community members to find providers willing to work with DHH people [65]. When they have providers who communicate directly in ASL, however, DHH ASL-users are more likely to receive preventive services [96] and be engaged in their healthcare [97].

### 3.2. Need and the Care-Seeking Decision

Health status, lifestyle factors, and acute experiences may lead to a health issue that serves as a catalyst to seeking care. At this point, DHH patients are faced with a decision: to use ED or acute care services, contact their usual provider, or delay care-seeking. This decision is driven primarily by the patient’s evaluation of their need to engage in healthcare. In the Andersen Model, this construct is called “perceived need” compared to “evaluated need,” which is based on medical provider assessment. The literature to date, however, shows that patients engage in a sophisticated decision-making process, evaluating their symptoms, their personal and social circumstances, and seeking advice from their social network, before seeking care [38,94,98,99]. Therefore, in the model, this construct is renamed “patient evaluated need.” The quality of patient evaluated need, including alignment with provider evaluated need, is based heavily on individual and community-level predisposing, enabling, and reinforcing factors. Patients with higher health literacy, access to financial and transportation resources, and more resourceful social networks may have more timely and higher quality evaluations. (This conceptualization of “patient evaluated need” aligns with Andersen’s description that “perceived need” should be primarily explained by knowledge, health beliefs, and social characteristics.). 

A DHH patient’s self-evaluation may lead them to (1) treating themselves or waiting-and-seeing if the health problem is self-resolving, (2) contacting and/or seeing a provider before using ED services, or (3) accessing care through the ED. Seeking care from a usual provider requires that the DHH patient have an existing patient-provider relationship and beliefs consistent with the use of primary care services for acute conditions. For example, in non-DHH samples, seeking ED care is related to patient beliefs and expectations including factors such as ED convenience [98,100], reduced delay in diagnosis and treatment [38,101], and a belief that using primary care for emergent conditions is inappropriate [98,99]. In addition, the environment must be enabling to the use of healthcare. This includes (1) a provider’s schedule availability [15,96,100,101], (2) the health system infrastructure’s accessibility concerning the DHH patient’s transportation resources [38,98,102], and (3) the patient having the financial resources necessary to access care [15,100]. Furthermore, communication accessibility for DHH ASL-users during emergent situations is likely to be subpar outside of the ED setting: it may be difficult getting an interpreter on short notice [65,97]. Therefore, in most cases, seeking ED care is likely to be due to perceived and actual difficulty accessing care and communication outside of the ED setting. 

If a patient receives a provider’s evaluation outside the ED, they may be directed to use the ED. In some cases, patients present to the ED based on previous discussions with their usual providers directing them to use the ED in the presence of a specific symptom [45,94,98,100]. A provider’s comfort with directing patients to the ED versus the clinic is influenced by their knowledge of the patient’s clinical history, condition progression, and social resources [98]. In addition, patients may forgo seeking healthcare outside the ED setting and go directly to the ED for care. At this point, DHH patients may consider ED quality indicators (e.g., wait times and previous communication experiences) to determine the specific ED location in which to seek care [94]. Once in the ED, the patient may receive a provider’s evaluation of their condition, which impacts treatment and diagnostic procedures. A patient’s access to a provider’s evaluation of their health condition contributes to modifying DHH patient skills, knowledge, and health beliefs. For example, a DHH patient with multiple chronic health conditions may have historically used the ED every time their condition acutely worsened. From the DHH patient’s perspective, seeking care was necessary for their condition and a precursor to a patient evaluated need of in-patient admission. After years of ED visits leading to in-patient care without receiving feedback from providers, a specialist provider may intervene to affirm the patient’s evaluation of the need to seek care and to teach the patient to, if possible, contact their office directly before seeking ED care. The patient now understands that they can be assessed and admitted directly from the specialist’s clinic, without using the ED. Without receiving the provider’s evaluation and education, the DHH patient may have continued to seek ED care.

### 3.3. ED Care Processes, Discharge, and Revisit

Once a DHH patient enters the ED, there is a strong convergence of patient and non-patient (i.e., contextual, health system, interpreter, and provider) factors occurring during the ED care process. This process represents the patient’s time in the ED, receiving diagnostic tests, diagnosis, treatment, and later being discharged from the ED. It is well established that hospital infrastructure and ED burden influence the care process of patients in the ED [103]. These non-patient factors, in addition to patient behavior, influence ED provider emotions [103]. The link between an ED provider’s emotions and patient care is important when considering the experience of DHH patients. In a qualitative study focused on ED communication experiences of DHH ASL-users, patients reported self-advocating and becoming frustrated with providers who were prescriptive with communication accessibility [94]. Challenging providers’ authority and power may lead to a provider being stressed or frustrated, and result in poorer patient–provider trust and poorer patient outcomes [65,103,104].

A provider’s training, beliefs, and knowledge about DHH people also influences the treatment process. Awareness of a DHH patient’s social resources and current access to healthcare should prompt ED providers and social workers to meet care needs. An understanding of prevalent issues, such as language deprivation, is also necessary: in some cases, DHH patients presenting to the ED exhibiting symptoms of language deprivation syndrome may be mischaracterized as experiencing psychosis [22]. This is noteworthy because healthcare providers endorse a medicalization of the DHH experience. For example, nurses endorse audist beliefs including believing deafness is a disorder needing correction and that all DHH people should wear hearing aids [95]. This may lead providers to focus on the condition of being DHH as a root cause (e.g., blaming the patient for their condition) rather than systems of oppression (e.g., audism) working against the DHH patient. 

As described in the next section, DHH patients are at high risk of receiving inaccessible, ineffective communication in healthcare settings; this is important to consider when the patient is admitted to an inpatient unit from the ED. DHH ASL-users in inpatient settings may not receive access, comparable to that of non-DHH patients, to diagnostic and treatment decisions or be engaged in shared decision-making [105]. 

DHH patients, like non-DHH patients, report difficulty at discharge, whether leaving the ED or an inpatient unit. This is due to a variety of factors including lack of discharge instructions in accessible language (e.g., typically provided in jargon-riddled English) [94], feeling rushed [38,94], and lacking linkage to accessible outpatient resources [38]. Each of these is moderated by the predisposing, enabling, and reinforcing factors within the model. DHH patients with adequate health literacy and/or English proficiency, resourceful social networks, or high engagement in healthcare may experience more effective discharge and post-discharge treatment plan adherence. Those without these protective factors, however, may experience discharge failure. 

As defined in the outcome definition of ED revisits, discharge failure occurs when patients do not understand and do not have the resources to adhere to their discharge instructions and treatment plan. Discharge failure is one of the causes of an ED revisit within an acute timeframe (e.g., 9 days). Discharge failure, however, is not the sole factor contributing to a revisit. The literature to date indicates the importance of patient beliefs and expectations of the ED care process. A patient who is dissatisfied with the initial ED encounter’s care processes, including the diagnostic procedures and treatment decisions, is likely to return to the ED [38]. Patients, as well as their social networks, may also be concerned when experiencing pain or a worsening of their condition [38]. In the context of the DHH experience, a revisit may be more likely: DHH patients who are denied communication access are systematically discouraged from being engaged in their ED care, leading to a misalignment between the care experience and patient expectations, and then patients are not provided accessible discharge instructions [94]. These factors are strongly related to the ED communication context. 

### 3.4. ED Communication Context

An important consideration for the ED care process for DHH patients is the communication that occurs between patients and providers, and internally between care staff. Communication is fundamental to an effective and efficient care process that will improve patient health; therefore, the process of care is encapsulated within the “ED/Hospital Communication Context.” In the U.S., several federal laws (e.g., Americans with Disabilities Act and the Patient Protection and Affordable Care Act) ensure the right of DHH patients to have effective communication access in healthcare settings. However, a qualitative study of DHH ASL-users’ experiences in the ED found that patient-provider communication was suboptimal [94]. Care team members failed to provide on-site ASL interpreters, and computer-based interpreting systems (i.e., Video Remote Interpreting (VRI)) are frequently subject to user and technical difficulties preventing the efficacy of VRI-mediated communication [94,106]. In fact, in a large national sample of DHH ASL-users (conducted between 2016 and 2018), 59% of those who used VRI services in the past year indicated they were unsatisfied with the experience [107]. This dissatisfaction is likely to be due to both technological interference (e.g., poor connection) and the skillset of the VRI interpreter [94,107].

Poor communication between patients and providers has contributed in part to providers’ limited training and experience in working with DHH patients leading to incorrect assumptions of patient communication modalities [65,95,97,108], and the health system’s unwillingness to accommodate DHH patients. For example, providers may hold beliefs that lipreading or written communication is effective, when DHH ASL-users report that it is not [94]. Delays in receiving effective communication may increase ED LOS; in some cases, patients have waited over eight hours for communication access in the ED [94]. Health system factors also influence effective communication, including policies that empower inexperienced care providers to make authoritative decisions about communication accommodations a DHH patient receives. For example, in a lawsuit where DHH ASL-using patients requested interpreters in the ED, hospital policy empowered healthcare workers (e.g., physicians and nurses) to make decisions regarding the provision of interpreters and other communication accommodations; the healthcare workers wrongly determined that interpreters were not needed [105]. 

In the presence of health system policies supportive of staff providing interpreters and other communication aids (e.g., captioning), effective communication is then also impacted by (1) the availability of the service within the region, and (2) the quality of the service. For example, the demand for interpreters in smaller cities may be significantly higher than the supply of interpreters available. In some cases, medical interpreters come from larger metropolitan areas hours away [94]; this may lead to ED administrators and staff perceiving an interpreter request as unreasonable. If an interpreter is available and is provided, the quality of the interpretation directly affects patient–provider communication. Therefore, the interpreter’s preparedness, knowledge of medical terminology and processes, and knowledge of methods to support patient–provider communication is paramount {Citation}. The interpreter’s professionalism is also considered: DHH ASL-users report that interpreters may engage in behavior detrimental to their communication access including being judgmental and disempowering DHH ASL-using patients from self-advocating [94]. 

## 4. Applying the Model for Hypothesis Generation, Research, and Practice

In developing this conceptual model, it was clear that the DHH population’s healthcare utilization behavior, including the barriers and facilitators to ED utilization, have not been widely studied. Existing research is largely descriptive or focuses on the role of communication access when accessing healthcare. Rightly so, the influence of the communication context cannot be overstated as communication access is pivotal for DHH patient engagement, informed consent, shared decision-making, and adherence to treatment and discharge plans. 

The factors specified in the model may be well established in health behavior and ED outcomes, generally. However, these factors have not been largely studied among the DHH population. Unlike the wealth of research available on English-speaking, non-DHH, or other priority patient populations, the field lacks research examining the extent of these predisposing, enabling, and reinforcing factors, and how these socio-behavioral antecedents can be modified in the DHH population. This impacts the development of DHH patient-centered healthcare and community-care models that may improve DHH patient outcomes. Therefore, more resources should be allocated to work with this priority population, applying this model for hypothesis generation, future research, and informing health promotion program planning and policy advocacy.

This conceptual model provides a foundation for research in DHH patient-centered outcomes research. The DHH population, and their health outcomes, are of central interest to funding agencies including the AHRQ and Patient-Centered Outcomes Research Institute [109]. Therefore, research may focus on both socio-behavioral antecedents and healthcare/ED utilization outcomes such as:

- Patient predisposing: The development of health beliefs and social norms for DHH patients, specifically group dynamics among DHH ASL-users and non-DHH people.
- Patient reinforcing: Mediating and moderating factors of how provider education to DHH patients influence knowledge and skills development.
- Non-patient enabling: The impact of health policy (e.g., Medicaid expansion) on reducing DHH patient health inequities.
- Non-patient enabling and reinforcing: How DHH patient advocacy for effective communication impacts ED providers’ perception of the patient.
- Health service outcomes: Cost-effectiveness studies to identify the impact of preventing chronic health conditions among DHH people to justify resource allocation to health promotion programs.
- ED outcomes: How interpreter provision accelerates or delays ED length of stay for DHH ASL-using patients.
- ED outcomes: How the communication context influences patient safety events and diagnostic delays among DHH patients.

Research should, however, consider the DHH person as more than just their DHH status. The social determinants of health, contexts in which a DHH person exists, and related social risk factors and social needs do not guarantee that a DHH person engages in health-compromising behavior. It is essential to holistically consider all aspects of a DHH person’s life including their access to health-promoting resources (e.g., social networks, insurance) and their experience with other fundamental causes (e.g., racism and socioeconomic position).

This model may also serve as a foundation for quality improvement and health promotion intervention development, including dissemination and implementation science and policy advocacy. Our model highlights the shared role of patient and non-patient factors affecting DHH patient ED utilization, and the abundance of enabling factors. Health promotion specialists can use this model to identify targets for interventions and quality improvement programs, and work with patients and other stakeholders to develop health-promoting programs. These targets could include provider and family education programs to increase DHH child exposure to visual languages to reduce language deprivation and associated long-term outcomes; development of healthcare navigation and care coordination programs for DHH patients; and improving the provision of effective communication with DHH patients. In applying this model, however, it is important to understand the relative importance and changeability of each of the constructs before developing programs [43]; this includes ensuring a focus on the structural issues affecting DHH patients, instead of allocating resources solely to individual-level programs. 

### 4.1. Assumptions

Based on the perspectives of the ecological and educational framework (framed in the SEM and PPM), there are several inherent assumptions of this model. First, applying an ecological framework requires recognition of the powerful effects of social and environmental structures. Therefore, the structural oppression of DHH individuals at the societal level must be addressed to achieve health equity; health equity will not occur by solely investing in individual-level behavior change programs. 

An additional assumption underlying the model is the incorporation of a transformative paradigm that focuses on increasing social justice when working in culturally diverse settings [110,111]. With the need for social justice for DHH people and recognition that DHH people are experts in their lived experiences, researchers are compelled to promote a transformative paradigm that includes Deaf epistemologies and a CritDeaf lens (i.e., critical theory applied to the DHH experience). The transformative paradigm calls for non-DHH researchers to recognize the power they bring to the research process and listen to community members [111]. This aligns with Deaf epistemologies, which unequivocally acknowledge DHH people as the authority on DHH community knowledge and lived experiences [112]. Therefore, research that does seek to extend this model should be community-focused, acknowledge the authority of DHH people as experts, and include community members and researchers who are DHH [113]. Consequently, in affirming Deaf epistemologies and ontologies, researchers and program practitioners need not be concerned if community-indicated concerns are not yet supported by conventional scientific research. Knowledge exists regardless of if it exists in the research literature. Therefore, concerns should be added to the model while waiting for community knowledge to be represented in the scientific literature. 

Lastly, the Andersen Model and PPM recognize the feedback loops that occur when patients engage in programs and services [42,43]. Patients, providers, and healthcare environments are complex adaptive systems that learn from their behavior, modifying future antecedents and influencing the performance of the system (i.e., reciprocal determinism) [41,43]. Therefore, the final part of the model is the central outcomes influencing predisposing, enabling, and reinforcing factors. This occurs regardless of the final ‘outcome’ of the model. If a patient chooses not to seek care, they learn from this experience; similarly, their future behavior is influenced by their satisfaction and access to communication when receiving ED care.

### 4.2. Limitatiions

We do not explicitly state hypothesized paths between antecedents and primary outcomes in the model. Existing, overarching paths are informed by the Andersen Model and PPM [43]. However, direct mediation and moderation paths, informed by existing health behavior theory, should be tested to understand how predisposing, enabling, and reinforcing factors interact and influence overall health behavior and ED-specific outcomes among DHH people. When considering the model’s scope and the assumption that inequities exist primarily due to audism, we do not contend that this model is exhaustive, nor does it fully account for the complexity of the widespread historical and present-day structural oppression of DHH individuals. 

Additional limitations are grounded in the study’s methodology, namely (1) lack of reproducibility, (2) potential misspecification, and (3) construct underrepresentation or exclusion. As critical reviews are non-systematic, it is impossible to replicate our search strategy; however, this method was the most appropriate for our research aim. Due to the overarching classification of construct domains, some sub-constructs may be mis-specified within the predisposing, enabling, and reinforcing domains. For example, health literacy is a multi-dimensional construct consisting of skills (enabling) and knowledge (predisposing). Relatedly, it is possible that some constructs were unintentionally excluded or underrepresented in the model. Lastly, we do not provide specific methods to measure the model constructs. This is in line with methodological recommendations not to provide operational definitions during model construction, as measurement decisions should be justified for individual studies [17,20]. Despite these limitations, this model was based on prior theories and models for health services research and health promotion and should be useful to guide future research in DHH patient ED utilization. However, the model should be revised iteratively as the evidence base on DHH patient health and ED utilization continues to expand.

## 5. Conclusions

DHH patients are a priority population yet remain underserved and understudied in health services research. Existing research identifies disparities in ED utilization among DHH patients. In this paper, we applied a critical review research methodology to identify socio-behavioral antecedents influencing DHH patient ED utilization. This model indicates the importance of enabling factors, i.e., skills, resources, and policies, for promoting DHH patient health, and underscores the role of barriers to healthcare navigation and utilization exacerbated by structural oppression. The Conceptual Model of Emergency Department Utilization Among Deaf and Hard-of-Hearing Patients should be used in research, health promotion and public health practice, and quality improvement programs to improve methodological rigor and, consequently, health equity for this population. 

## Data Availability

Data available are from the cited sources in this review.

## Appendix A

**Table A1:** Patient-level constructs and supporting evidence of the proposed conceptual model.

Construct	Definition	Citations
Predisposing: Beliefs regarding need of an interpreter ^a,b,d^	DHH ASL-users may assess their need for an interpreter during a medical encounter based on the complexity of the situation and the expected amount of communication needed. This construct may align strongly with perceived threat (as described in the Health Belief Model) of not having an interpreter.	[65,94]
Predisposing: DHH-specific demographic and cultural characteristics ^a,b,d^	Characteristics that are unique to DHH individuals, such as: Age of onset of deafness Deaf school education DeafBlind identity Deaf cultural factors Language modality	[15,26,90,94,105,114,115]
Predisposing: Demographic and cultural characteristics ^a,c,d^	General demographic characteristics, including: Age Education Employment Race and ethnicity English proficiency General cultural factors	[15,26,38,94,100,101,105,116,117]
Predisposing: Early childhood language exposure and information access ^b,d^	Describes the DHH patient’s early childhood language environment, including experience of language deprivation, and access to incidental learning and indirect communication.	[58,60,63,64,65,115,118,119,120]
Predisposing: Expectancies of ED care processes ^a,c^	Describes patient expectations of using the ED including: Affordability of ED services relative to other sources of care. Comparative beliefs of quality of care (e.g., based on diagnostic and treatment resources, provider availability) in ED versus non-ED settings. Expectation of condition/symptom improvement after the initial ED visit. Perception of wait times of sources of care (e.g., ED, urgent care, or primary care). Perceptions regarding convenience of using the ED versus a usual care or urgent care provider. Satisfaction of initial ED visit chief diagnosis and treatment processes.	[38,94,98,99,100,101]
Predisposing: Healthcare system beliefs, use, and experience ^b,d^	General healthcare beliefs, utilization, experiences and satisfaction including: Awareness or belief of limited outpatient availability for follow-up. Belief that PCP is not available for urgent appointment. Perception of the role of a PCP/usual provider for providing “routine care” rather than acute or urgent care. Satisfaction with regular source of care.	[24,38,98,99]
Predisposing: Health beliefs ^b,d^	Beliefs and cognitive appraisals related to health behavior such as: Fear Perceived benefits and barriers Perceived threat (i.e., severity and susceptibility) Self-efficacy to engage in health-promoting behavior	[38,41,98,99,121]
Enabling: Access to a regular provider ^a,b^	Access to a usual provider that can provide an alternative source of care for patients seeking ED services, or provide health-promoting information.	[65,90,94]
Enabling: Access to resources for medical information ^b^	Provision of/access to health education/promotion materials (e.g., captioned videos with ASL) that educate patients on chronic condition management, risks, treatment options, and prevention.	[65,114]
Enabling: Assistive hearing technology ^b,d^	A patient’s use of assistive hearing technology (e.g., hearing aids) and if/how it augments their communication access.	[9,77]
Enabling: Behavioral capability and self-activation for health management ^b,d^	Patient’s understanding and skill set to do a behavior, including being involved in their healthcare decisions. This construct describes factors such as: Behavioral capability to engage in health-promoting behavior as described in the Social-Cognitive Theory. Experiences with patient activation in adolescence and adulthood.	[41,65,77]
Enabling: Behavioral capability and skills of self-advocacy for communication ^a,b^	Describes a DHH patient’s skills for advocating for communication access, including: Confidence in self-advocating and knowing the law. Knowledge of accessibility law and rights. Knowledge and skills modifying environments for access. Resilience and persistence when faced with negotiation and denial of access. Skills with negotiating interpreter access.	[65,94,97,114]
Enabling: Financial considerations ^a,b,c^	Financial considerations describe a patient’s cognitive processes and available resources regarding: Balance of the cost of engaging in healthcare with living expenses. Cost of visiting the ED relative to other sources of care, including payment flexibility for ED care. Income. Insured status. Perception of the quality of ED care relative to cost, compared to other sources of care.	[15,16,23,45,98,100,101,114]
Enabling: Health literacy and health navigation ^a,b,c,d^	Abilities related to the overall construct of health literacy and health navigation including: Condition specific knowledge Family health history knowledge Health literacy Health navigation Health insurance literacy Health insurance navigation Knowledge and skills of compensatory strategies (e.g., advance directives) to mitigate systemic barriers within healthcare.	[15,24,64,65,93,98,99,101,114,122]
Enabling: Limited use of ED because of restrictions ^c^	Idiosyncratic restrictions that prevent ED utilization, such as caregiving responsibilities to a family member or pet.	[98,99]
Enabling: Transportation access ^c^	A patient’s access to transportation with consideration of their distance to care. Transportation access is influenced by access to social and economic resources.	[38,98,101,102,122]
Reinforcing: Audism ^b,d^	Individual “beliefs and behaviors that assume the superiority of being hearing over being Deaf” [56], an institutional “system of advantage based on hearing ability” [56], and “a metaphysical orientation that links human identity with speech” (Bauman, 2004, p. 245). Examples of audism include: Banning the use of visual language modalities (e.g., ASL) in favor of oral-aural education. Conflating a DHH person’s intellect based on their language modality or ability to use spoken language. Failing to see a DHH person as more than their hearing ability. Forcing a DHH person to conform to hearing society	[56,59,123]
Reinforcing: ED provider response of utilization ^c^	An ED provider may respond in different ways to a patient’s use of the ED, particularly when it is deemed medically non-urgent. This response may include: Educating patients when it is “appropriate” to use the ED Empowering patients to manage conditions prior to ED utilization Tailoring communication based on a patient’s social resources and access to care Withholding education and opinion regarding the ED visit	[124]
Reinforcing: Exaggeration of symptoms ^b^	Healthcare encounters when patients exaggerate or fake symptoms, such as complaining about chest pains when there are none, to change the process of care (e.g., getting faster care).	[65]
Reinforcing: Family and social network factors ^a,b,d^	Family members’, friends’, and others in the patient’s social network influence on health behavior and healthcare-seeking including: Deaf person’s sense of belongingness influencing information seeking. Explaining treatment plans. Providing information on providers or clinics/hospitals who are friendly and accessible to DHH patients.	[38,41,64,65,94,115]
Reinforcing: Power differential with patients, providers, and interpreters ^a,b,d^	Describes the power differential between patients and their healthcare providers and/or ASL interpreters. ASL interpreters usurping a DHH patient’s ability to self-advocate. DHH patients feeling unable to confront providers during miscommunications or misunderstandings. DHH ASL-users perceiving their interpreter request as a challenge to the medical provider’s authority. Patients feeling uncomfortable challenging or renegotiating a provider’s treatment plan.	[65,94,95,123]
Reinforcing: Provider advice to use ED ^a,c^	Communication received from usual care providers for patients to initially use or revisit an ED during specific situations (e.g., time of day, experiencing specific symptoms).	[38,94,98]
Reinforcing: Quality of patient interactions, education, and communication ^a,b,c^	Concepts regarding the quality of the provision of patient education and patient satisfaction with their patient-provider relationship, such as: Effective communication between patient and provider. Provider provides information regarding diagnostic and treatment decisions. Quality of patient-provider relationship including provider follow-up.	[22,24,94,114,116,125,126]
Reinforcing: Social network advice to use ED ^a,c,d^	Recommendations to seek care at (or revisit) an ED from individuals within the patient’s social network including friends or family members, particularly those who have experience with the condition or healthcare system. These recommendations may be unsolicited or solicited.	[38,45,94,98]
Reinforcing: Trust and working alliance with providers and interpreters ^a,c,d^	Describes trust between patients, providers, and interpreters including: DHH patient trust and working alliance with the interpreter. DHH patient trust and working alliance with the provider. Medical provider trust and working alliance with the interpreter. Working history between patients, providers, and interpreters.	[38,65,94,98,101,114]

^a^ Evidence from investigations of ED outcomes among DHH patients; ^b^ general health outcomes among DHH patients; ^c^ ED and health outcomes among other patient populations; and, ^d^ theory-, practice-, or community-informed sources.

**Table A2:** Non-patient-level constructs and supporting evidence of the proposed conceptual model.

Construct	Definition	Citations
Predisposing: Awareness and beliefs of Deaf culture and communication modalities ^a,c,d^	Describes a healthcare provider’s awareness of Deaf culture and accessible communication modalities including: Beliefs regarding the medicalization of a DHH person (e.g., the need for a DHH patient to be “fixed”). Knowledge of how to effectively work with signed language interpreters. Knowledge of how to facilitate communication for DHH patients. Openness to accommodating the DHH patient based on their requested modality. Perceptions of the efficacy of alternative communication modalities (e.g., lipreading and written communication). Recognition of heterogeneity of DHH experiences and involvement in Deaf culture.	[15,65,94,95,97,108,126]
Predisposing: Awareness of ADA policy and interpreter provision ^a,b^	Describes a healthcare provider’s awareness of their healthcare system’s accommodations policy and who is responsible for providing accommodations. Domains include: Awareness of provider/health system (not the patient’s) responsibility for interpreter provision. Knowledge of payment processes for auxiliaries (e.g., signed language interpreters). Knowledge of federal and state law to provide effective communication. Resistance to providing interpreters due to perceived exemptions (e.g., costs).	[15,94,95,105,108,126]
Predisposing: DHH-specific demographic and cultural characteristics ^b,d^	Describes characteristics of interpreters or medical providers who may be DHH including: Age of onset of deafness Deaf school education Deaf cultural factors (e.g., Children of Deaf Adults interpreters) Language modality	[127,128,129]
Predisposing: Demographics and cultural characteristics ^c,d^	General demographic characteristics of interpreters and medical providers, including: Age Education Race and ethnicity General cultural factors	[130,131]
Enabling: Access to healthcare advocates ^a,b,d^	Access to advocates for navigation or communication access that remove barriers and stressors for the patient. Relevant characteristics include: Advocate knowledge and skills when working and communicating with DHH patients – including fluency in ASL. Advocate’s integration within the healthcare system.	[94,114]
Enabling: Access to specialized DHH/DB services ^b,d^	Access to specialized DHH and DeafBlind services which can provide: Advocacy Certified Deaf Interpreters DeafBlind Support Service Providers/CoNavigators Social support and linkage to resources	[26,94,114]
Enabling: Availability and quality of ASL interpreters ^a,b^	Describes the availability of ASL/English interpreters and the quality of those interpreters including: Available interpreter control options to reduce the impact of interpreting demands. Health system employment arrangement with interpreters. Interpreter ASL and English fluency and cultural mediation skill. Interpreter professionalism. Knowledge and skillset interpreting medical terminology. Supply of interpreters with respect to local demand. Time of day or day of week.	[15,22,24,65,94,97,126,132,133]
Enabling: Community environment ^c,d^	Community physical and social environment factors including: Access to health-promoting resources (e.g., food, sidewalks, parks, etc.) Air and water quality Community distance from sources of care Housing quality Neighborhood income/poverty Neighborhood violence	[42,46,101]
Enabling: Competency working with DHH patients and interpreters ^a,b,d^	Describes a provider’s skillset working with DHH patients and interpreters including: Appropriate use of an interpreter (e.g., not asking the interpreter to ‘tell the patient’) Appropriate use of video relay service (VRS) Behavioral capability of requesting interpreter services Provider ASL fluency Skills with setting up and using VRI	[65,94,95,96,97]
Enabling: ED infrastructure and burden ^a,b,c^	ED system factors including:Consulting physician attitudes and availability ED census and over-crowdingUnderstaffing and turnover of ED staff and providers	[65,94,103,104]
Enabling: Emotional regulation ^c^	An ED provider’s strategies to regulate their emotions which impact their provision of patient-centered care, including: Cognitive reappraisal Distraction Emotional suppression Support seeking Rushing through communication with patients.	[103]
Enabling: Health insurance coverage of hearing technology ^b,d^	The cost of accessible hearing technology (e.g., hearing aids) – commonly not covered by insurance – is prohibitive to their usage.	[9,114]
Enabling: Healthcare infrastructure ^b,c,d^	Describes the local healthcare infrastructure including: Appropriate staffing Availability of primary care and specialty providers (e.g., clinic schedules) Availability of hospital beds Clinical resources for diagnosing and treating patient conditions Distribution of providers relative to patient need Local designation of a Healthcare Professional Shortage Area	[38,103,134]
Enabling: Health system policy for accessibility ^a,b^	Hospital/health system characteristics and accessibility polices that influence provider and patient communication by: Allowing providers and nurses to be the authority on providing communication aids. Dictating the type of accommodation to provide DHH patients (e.g., Video Remote Interpreting).	[94,105,116,126]
Enabling: Legal mandates on interpreter provision ^b,d^	Federal, state, and local policies regarding interpreter provision and the quality of interpreter services.	[65,135,136]
Enabling: Policies on community and environmental health ^c,d^	Federal, state, and local policies (e.g., public health law) that impact community and environmental health.	[41,101]
Enabling: Provider skill and quality ^b,d^	Describes a medical provider’s overall skillset and quality including: Provider bedside manner and communication skills Technical quality of the medical care provided	[114]
Reinforcing: Anticipated or actual punishment for quality issues ^a,b,d^	Salience of provider and interpreter professional malpractice, and/or accessibility violation dispute mechanisms on provider, interpreter, and health system behavior, including: Fear of lawsuit, ethics, or accessibility law compliance grievance. Previous lawsuit or settlement agreement mandating accessibility provisions (e.g., availability of interpreters, availability of other auxiliary aids, training).	[105,116,126]
Reinforcing: Audism ^b,d^	Individual “beliefs and behaviors that assume the superiority of being hearing over being Deaf” [56], an institutional “system of advantage based on hearing ability” [56], and “a metaphysical orientation that links human identity with speech” (Bauman, 2004, p. 245). Examples of audism include: Banning the use of visual language modalities (e.g., ASL) in favor of oral-aural education. Conflating a DHH person’s intellect based on their language modality or ability to use spoken language. Failing to see a DHH person as more than their hearing ability. Forcing a DHH person to conform to hearing society.	[56,59,123]
Reinforcing: DHH patient’s advocacy and communication behavior ^a,b,c^	A DHH patient’s behavior during healthcare encounters and its related outcomes including: A provider being exposed to DHH people who use both spoken and signed languages influencing their perspective. The influence of advocacy behavior on the provider’s perspective of the patient (e.g., seeing requests for interpreters as disruptive or demanding).	[65,94,103,104,132]
Reinforcing: Power differential between patients, providers, and interpreters ^a,b,d^	Describes the power differential between patients and their healthcare providers and/or ASL interpreters. ASL interpreters usurping a DHH patient’s ability to self-advocate. DHH patients feeling unable to confront providers during miscommunications or misunderstandings. DHH ASL-users perceiving their interpreter request as a challenge to the medical provider’s authority. Dual relationship with DHH medical providers and/or ASL interpreters. Patients feeling uncomfortable challenging or renegotiating a provider’s treatment plan.	[65,94,127]
Reinforcing: Trust and working alliance between patients, providers, and interpreters ^a,b,c^	Describes trust between patients, providers, and interpreters including: DHH patient trust and working alliance with the interpreter. DHH patient trust and working alliance with the provider. Dual relationship with DHH medical providers and/or ASL interpreters. Medical provider trust and working alliance with the interpreter. Working history between patients, providers, and interpreters.	[38,65,94,98,101,114,127]

^a^ Evidence from investigations of ED outcomes among DHH patients; ^b^ general health outcomes among DHH patients; ^c^ ED and health outcomes among other patient populations; and, ^d^ theory-, practice-, or community-informed sources.
